# Sepsis assessment and management in critically Ill adults: A systematic review

**DOI:** 10.1371/journal.pone.0270711

**Published:** 2022-07-01

**Authors:** Mohammad Rababa, Dania Bani Hamad, Audai A. Hayajneh

**Affiliations:** Adult Health Nursing Department, Faculty of Nursing, Jordan University of Science and Technology, Irbid, Jordan; Bay Area Hospital, North Bend Medical Center, UNITED STATES

## Abstract

**Background:**

Early assessment and management of patients with sepsis can significantly reduce its high mortality rates and improve patient outcomes and quality of life.

**Objectives:**

The purposes of this review are to: (1) explore nurses’ knowledge, attitude, practice, and perceived barriers and facilitators related to early recognition and management of sepsis, (2) explore different interventions directed at nurses to improve sepsis management.

**Methods:**

A systematic review method according to the PRISMA guidelines was used. An electronic search was conducted in March 2021 on several databases using combinations of keywords. Two researchers independently selected and screened the articles according to the eligibility criteria.

**Results:**

Nurses reported an adequate of knowledge in certain areas of sepsis assessment and management in critically ill adult patients. Also, nurses’ attitudes toward sepsis assessment and management were positive in general, but they reported some misconceptions regarding antibiotic use for patients with sepsis, and that sepsis was inevitable for critically ill adult patients. Furthermore, nurses reported they either were not well-prepared or confident enough to effectively recognize and promptly manage sepsis. Also, there are different kinds of nurses’ perceived barriers and facilitators related to sepsis assessment and management: nurse, patient, physician, and system-related. There are different interventions directed at nurses to help in improving nurses’ knowledge, attitudes, and practice of sepsis assessment and management. These interventions include education sessions, simulation, decision support or screening tools for sepsis, and evidence-based treatment protocols/guidelines.

**Discussion:**

Our findings could help hospital managers in developing continuous education and staff development training programs on assessing and managing sepsis in critical care patients.

**Conclusion:**

Nurses have poor to good knowledge, practices, and attitudes toward sepsis as well as report many barriers related to sepsis management in adult critically ill patients. Despite all education interventions, no study has collectively targeted critical care nurses’ knowledge, attitudes, and practice of sepsis management.

## Introduction

Sepsis is a global health problem that increases morbidity and mortality rates worldwide and which is one of the most common complications documented in intensive care units (ICUs) [1]. About 48.9 million cases of sepsis and 11 million sepsis-related deaths were documented in 2017 worldwide [2]. Sepsis is an emergency condition leading to several life-threatening complications, such as septic shock and multiple organ dysfunction and failure [3]. Sepsis has negative physiological, psychological, and economic consequences. Untreated sepsis can lead to septic shock; multiple organ failure, such as acute renal failure [4]; respiratory distress syndrome [5]; cardiac arrhythmia (e.g. Atrial Fibrillation) [6]; and disseminated intravascular coagulation (DIC) [7]. Also, sepsis is associated with anxiety, depression, and post-traumatic stress disorder [8]. As for the financial burden of sepsis on the healthcare system, the cost of healthcare services and supplies for ICU critical care patients with sepsis is high [1]. In 2017, the estimated annual cost of sepsis in the United States (US) was over $24 billion [2].

Previous studies have shown that among nurses, misunderstanding and misinterpretation of the early clinical manifestations of sepsis, poor knowledge, attitudes, and practices related to sepsis, and inadequate training might lead to delayed assessment and management of sepsis [9–11]. Moreover, the limited numbers of specific and sensitive assessment tools and standard protocols for the early identification and assessment of sepsis in critical care patients leads to delayed management, therefore increasing sepsis-related mortality rates [10].

Critical care nurses, as frontline providers of patient care, play a vital role in the decision-making process for the early identification and prompt management of sepsis [11]. Therefore, improving nurses’ knowledge, attitudes, and practices related to the early identification and management of sepsis is associated with improved patient outcomes [12, 13]. To date, there remains a wide gap between the findings of previous research and sepsis-related clinical practice in critical care units (CCUs). Furthermore, there is no evidence in the nursing literature regarding nurses’ knowledge, attitudes, and practices related to the early identification and management of sepsis in adult critical care patients and the association of these factors with patient health outcomes. Therefore, summarizing and synthesizing the existing research on sepsis assessment and management among adult critical care patients is needed to guide future directions of sepsis-related clinical practice and research. Accordingly, this review aims to identify nurses’ knowledge, and attitudes, practices related to the early identification and management of sepsis in adult critical care patients.

## Materials and methods

The present review used a systematic review design guided by structured questions constructed after reviewing the nursing literature relevant to sepsis assessment and management in adult critical care patients. The authors (MR, DB, AH) carefully reviewed and evaluated the selected articles and synthesized and analyzed their findings to reach a consensus. This review was guided by the following questions: (a) what are nurses’ knowledge, attitudes, and practices related to sepsis assessment and management in adult critical care patients?, (b) what are the perceived facilitators of and barriers to the early identification and effective management of sepsis in adult critical care units?, and (c) what are the interventions directed at improving nurses’ sepsis assessment and management?

### Eligibility criteria

The review questions were developed according to the PICOS (Participants, Interventions, Comparisons, Outcome, and Study Design) framework, as displayed in Table 1.

**Table 1:** The construction of review questions according to PICOS framework.

Item	Description
Participants	patients aged 19 years and older who were admitted to critical care settings with sepsis, septic shock, or septicemia
Intervention	Training/educational interventions (e.g., regular lectures, simulations, algorithms, decision support tools, and sepsis protocol)
Comparison	No restriction was applied on the number or type of comparison group as the impact of the intervention could be determined. Comparison groups could include no intervention, standard protocol, and other types of intervention which was educational
Outcome	The primary outcomes of interest in this review were the effective assessment and prompt management of sepsis and nurses’ knowledge, attitudes, practice, perceived barriers, and enablers related to sepsis assessment and management. sepsis assessment and management could be assessed using either patient or nurse objective measures. Sepsis assessment and management were quantified as mean times required for sepsis recognition and treatment initiation, sepsis protocol adherence, and decline in mortality rate in-hospital sepsis-related complications. nurses’ knowledge, attitudes, and practice related to sepsis could be assessed using either nurse-reported tools or performance-based tests, while nurses’ perceived barriers and enablers could be assessed using nurse-reported tools.
Study Design	Experimental, quasi-experimental, description. Cross-sectional, observational, prospective, qualitative, and mixed methods

#### Inclusion criteria

The articles were retrieved and assessed independently by two researchers (MR, DB) according to the following inclusion criteria: (1) being written in English, (2) having an abstract and reference list, (3) having been published during the past 10 years, (4) focusing on critical care nurses as a target population, (5) examining knowledge, attitudes, and practices related to the assessment and management of sepsis, and (6) having been conducted in adult critical care units.

#### Exclusion criteria

Studies were excluded if they were (1) written in languages other than English, and (2) conducted in pediatric critical care units or non-ICU. Dissertations, reports, reviews, editorials, and brief communications were also excluded.

#### Search strategy

**Fig 1:**
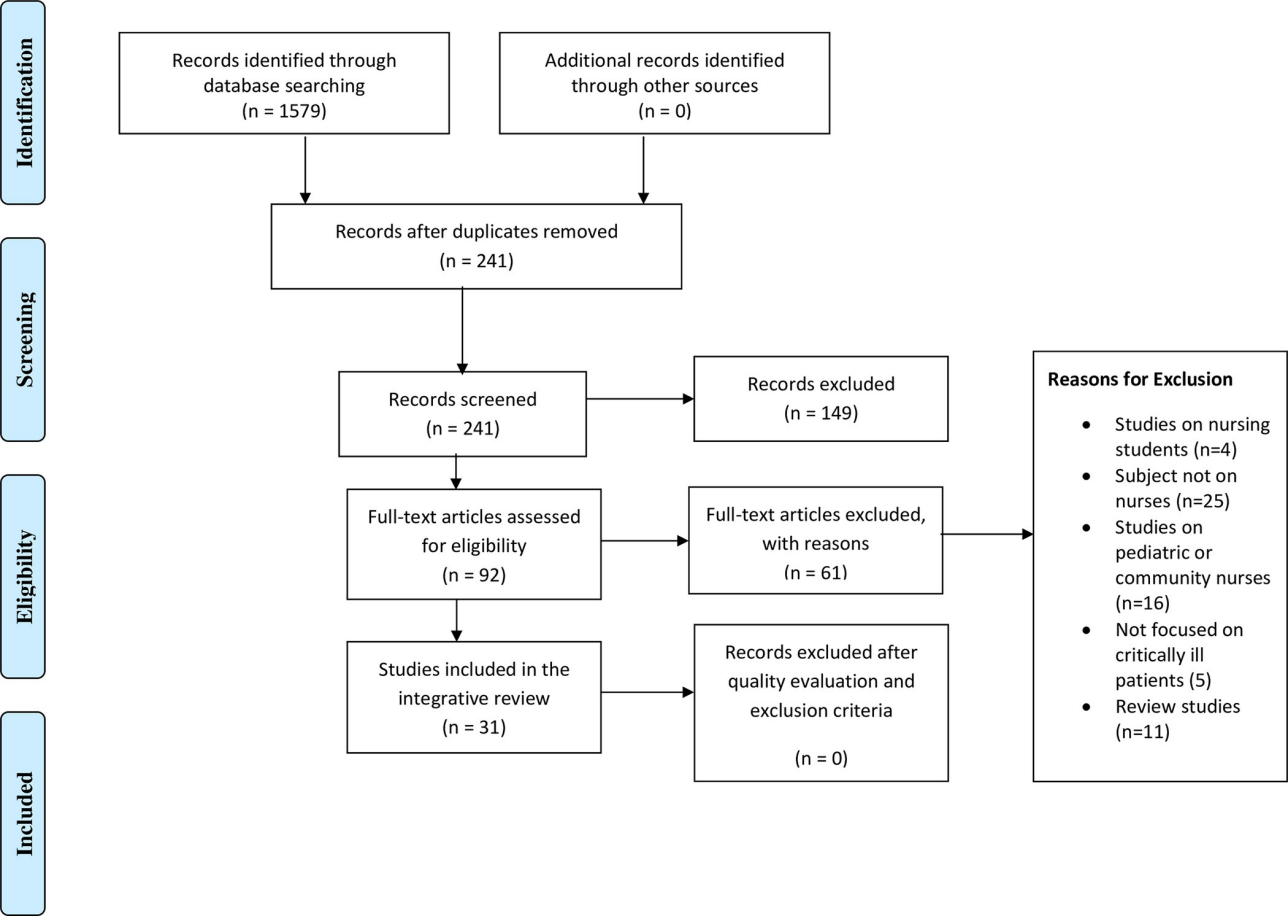
PRISMA flow chart.

An electronic search of the databases CINAHL, MEDLINE/PubMed, EBSCO, Embase, Cochrane, Scopus, Web of Science, and Google Scholar was conducted using combinations of the following keywords: critical care, intensive care, critically ill, critical illness, knowledge, awareness, perception, understanding, attitudes, opinion, beliefs, thoughts, views, practice, skills, strategies, approaches, barriers, obstacles, challenges, difficulties, issues, problems, limitations, facilitators, motivators, enablers, sepsis, septic, septic shock, and septicemia. The search terms used in this review were described in S1 File. The search was initially conducted in March 2021, and a search re-run was conducted in April 2022. The search was conducted in the selected databases from inception to 4/2022. The initial search, using the keywords independently, resulted in 1579 articles, and after using the keyword combinations, this number was reduced to 241 articles. Then, after applying the inclusion and exclusion criteria, the number of articles was reduced to 92. A manual search of the reference lists of the 92 articles was carried out to identify any relevant publications not identified through the search. The researcher (MR) used the function “cited by” on Google Scholar to explore these publications in more depth. The researchers (MR, DB) then screened the identified citations of these publications, applying the eligibility criteria. In case of discrepancies, the researchers (MR, DB) discussed their conflicting points of view until a consensus was reached. Then, after careful reading of the article abstracts, 61 irrelevant articles were excluded, and a total of 31 articles were included in this review. Fig 1 below shows the Preferred Reporting Items for Meta-Analysis (PRISMA) checklist and flow chart used as a method of screening and selecting the eligible studies.

### Data extraction

The following data were extracted from each of the selected studies: (1) the general features of the article, including the authors and publication year; (2) the characteristics of the study setting (e.g., single vs. multisite); (3) the sociodemographic and clinical characteristics of the target population, including mean age, and medical diagnosis (e.g., sepsis, septic shock, and SIRS); (4) the name of the sepsis protocol used, if any; (5) the characteristics of the study methodology (e.g., sample size and measurements); (7) the main significant findings of the study; and (8) the study strengths and limitations. All extracted data were summarized in an evidence-based table (Table 2). Data extraction was performed by two researchers (MR, DB). An expert third researcher (AH) was consulted to reach a consensus between the two researchers throughout the process of data extraction.

**Table 2:** Summary of the reviewed studies.

Study	Aim of the study	Design	LOE	Setting/Sample	Main findings	Strengths/Weaknesses
Delaney et al. (2015)	To determine the impact of an educational program on nurses’ assessment & management of sepsis	Quasi-experimental	III	82 ER nurses/ USA	There was a significant improvement in nurses’ knowledge & competency related to the early recognition & management of sepsis after the educational program.	**weakness**: use of self-report tools, purposive sample, homogeneity of sample. **Strength**: use of reliable and valid tool
Breen and Rees (2018)	To identify the barriers to the implementation of sepsis protocols	Cross-sectional	VI	108 nurses in ACS/UK	Nurses’ poor knowledge & poor ability to recognize sepsis during observation round were the main barriers to prompt sepsis management	**Weakness**: low response rate, heterogeneity of sample **Strength**: several geographical areas
Roney et al. (2020)	To evaluate the implementation of MEW-S in ACS	Quasi-experimental	III	139 nurses in ACS/ USA	Implementation of MEW-S led to a significant improvement in sepsis assessment & management, thus decreasing mortality rate by 24%	**Weakness**: one geographical site **Strength**: use reliable & valid tools
N. Roberts et al.(2017)	To identify the barriers to and facilitators of the implementation of the Sepsis Six at a case study hospital	Mixed method	VI	13 ER nurses /USA	The main barriers were insufficient audit & feedback, poor teamwork & communication, & insufficient training & resources. Main facilitators were good confidence in knowledge & skills & positive beliefs towards sepsis bundles	**Weakness**: one geographical site **Strength**: used mix methods design
van den Hengel et al. (2016)	To examine the factors influencing the knowledge & recognition of SIRS criteria & sepsis by ER nurses	Prospective -observational	IV	216 ER nurses from 11 hospitals/ Netherlands	ER nurses aged over 50 had significantly lower scores in knowledge related to sepsis criteria than did younger nurses. Nurses working in hospitals with 3 level ICUs had more knowledge than did nurses working in hospitals with levels 1&2 ICUs. The educational program improved nurses’ knowledge of sepsis.	**Weakness**: potential bias because multiple visits were made **Strength:** conducted in multi- center sites
Long et al. (2018)	To gain insight into clinical decision support systems-based alert and nurses’ perceptions	Cross-sectional	VI	43 ER nurses/USA	Using clinical decision support systems-based alert improved nurses’ decision-making related to sepsis, thus leading to better outcomes	**Weakness**: not validated questionnaire, conducted in single center **Strength**: used interactive survey to collect data
Jacobs (2020)	To determine if implementing the NDS protocol reduces ACT readmission among patients with sepsis	Quasi-experimental	III	238 patients with sepsis/ USA	Readmission rate among patients assessed & treated by NDS & who received early-goal directed therapy was reduced from 36.28% to 25% after 8 weeks. Nurses’ compliance with the intervention protocol was improved.	**Weakness**: the protocol used was not universally applied **Strength:** novelty of the study and use of protocol based on the golden criteria of the SSC
Amland et al. (2015)	To examine the diagnostic accuracy of two-stage clinical decision support systems for the early recognition & management of sepsis	Observational cohort study	IV	417 patients with sepsis/ USA	Nurses completed 75% of assessment and screening within one hour of notification. The decision support system led to the early identification and timely, quality, and safe sepsis care	**Weakness**: single center **Strength**: used sepsis alert with high positive predictive values
Delawder and Hulton (2020)	To test the effectiveness of sepsis bundle guidelines in the early assessment & treatment of sepsis.	Quasi-experimental	III	214 ER patients /USA	There was an improvement in the time to implement sepsis guidelines, except for antibiotic administration & blood culture collection. Mortality rate decreased from 12.45% to 4.55% but no differences in mortality rate based on age or gender	**Weakness**: single center **Strength**: used an interdisciplinary trained team & standard guidelines for sepsis
Manaktala & Claypool (2017)	To evaluate the impact of a computerized surveillance algorithm & decision support system on sepsis mortality rates	Quasi-experimental	III	58 patients in Huntsville hospital (tertiary care teaching hospital/ USA)	The system was sensitive & specific for sepsis identification & management & improved decision-making related to sepsis management. Mortality rate was reduced by 53% & readmission rate was reduced, with no effect on patient length of stay	**Weakness**: Small sample size **Strength**: used different methods to detect mortality rate related to sepsis
Harley et al. (2019)	To explore and understand ER nurses’ knowledge of sepsis & identify gaps in clinical practice related to sepsis management.	Qualitative	VI	14 ER nurses/ Australia	Nurses had poor knowledge, attitudes, & practices related to sepsis assessment & management. Barriers to sepsis management included high number & severity of sepsis conditions, nurses’ poor knowledge of sepsis, heavy workloads, & inexperienced ER doctors	**Weakness**: fatigue was a threat to internal validity, single center, & use of self-report tools **Strength**: used detailed face to face interviews
Yousefi et al. (2012)	To review the effect of an educational program on nurses’ knowledge, attitudes, & practices related to the identification & management of sepsis	Quasi-experimental	III	64 ICU nurses/ Iran	Nurses’ knowledge, attitudes, & practices were improved after the intervention	**Weakness**: Differences in ICU facilities and equipment made it difficult to generalize the findings **Strength**: used of valid & reliable tool
Nucera et al. (2018)	To assess knowledge and attitudes related to sepsis among ICU and non-ICU nurses and physicians	Quasi- experimental	III	11 different wards (ICU and non-ICU) in Italy	Nurses’ attitudes towards blood culture technique were poor & their knowledge of blood culture procedures & sepsis risks was good (>75%). Nurses had poor knowledge (<50%) of methods for the early identification, diagnosis, & management of sepsis. Their knowledge of sepsis improved after the intervention educational program	**Weakness**: Heterogeneity of the sample **Strength**: High response rate and zero attrition rate
Rahman et al. (2019)	To explore nurses’ knowledge & attitudes related to the early identification & management of sepsis	Cross-sectional	VI	120 ER in Malaysia	Nurses had poor knowledge of & neutral attitudes towards sepsis.	**Weakness**: single center & low validity **Strength**: detailed description of instruments
Storozuk et al., (2019)	To assess ER nurses’ knowledge of sepsis & their perspectives towards caring for patients with sepsis	Cross-sectional	VI	758 ER nurses/ Canada	Most nurses had poor knowledge of sepsis & SIRS definition, general knowledge, & treatment. Nurses were aware of the need to update their knowledge related to the early identification & timely management of sepsis to reduce complications	**Weakness**: single site **Strength**: the questionnaire used was based on the standard guidelines of the SSC
Gyang et al. (2015)	To evaluate the use of NDS for early sepsis identification	Observational pilot	IV	245 patients with sepsis in intermediate care settings/ USA	The NDS had 95% sensitivity and 92% specificity.	**Strength**: used a highly sensitive screening tool **Weakness**: one geographical site
El Khuri et al. (2019)	To assess the effect of EGDT in the ER on mortality rates related to sepsis and septic shock	Retrospective cohort	IV	290 patients with sepsis from one large tertiary hospital in Lebanon	There were no differences between the two groups in time & duration of vasopressor, antibiotics, and length of stay. The implementation of EGDT in the ER decreased the mortality rate from 47.6% to 31.7%. The most common cause of infection leading to sepsis was LRTI.	**Strength**: first study conducted in Lebanon **Weakness**: conducted in one site
Vanderzwan et al. (2020)	To apply a multimodel nursing pedagogy with medium fidelity simulation senarios for the early identification & management of sepsis	Quasi-experimental	III	All critical care nurses in an academic medical center/ USA	Nurses’ knowledge & competency related to the early identification & management of sepsis improved after simulation	**Weakness**: Only face validity was used to validate the questionnaire **Strength**: used multimodal in intervention
R. J. Roberts et al. (2017)	To evaluate nurses’ knowledge, attitudes, & perceptions related to antibiotic innitiation for patients with sepsis	Cross-sectional	VI	122 critical care nurses/ USA	Nurses had good knowledge related to defining septic shock & were aware of Aware of when to administer antibiotics. Lack of awareness of the importance of antibiotics initiation, lack of IV access, & the need for multiple medications rather than antibiotics were major barriers to sepsis management	**Weakness**: Self-selection and single center **Strength**: valid tools
McKinley et al. (2011)	To compare between paper protocols & computerized protocols for standarizing sepsis decision-making	Quasi- experimental	III	948 ICU nurses in an academic tertiery hospital in the USA	The computerized protocol led to quicker antibiotic administration, blood culture collection, and lactate level checking as compared to the paper-based protocol. The computerize protocol had 97% sensitivity & 97% specificity to the standardized & rapid implementation of evidence-based treatment guidelines of sepsis	**Weakness**: Technical issues in implementing the protocol **Strength:** the intervention was applied over a long period of time
Drahnak et al. (2016)	To assess the impact of an educational program on nurses’ knowledge, perceptions, & attitudes related to sepsis	Quasi-experimental	III	680 ICU & ER nurses/ Pennsylvania, USA	Knowledge of sepsis was improved after the educational program. There was significant improvement in nurses’ ability to identify patients with sepsis	**Weakness**: high attrition rates **Strength: used** standard guidelines for sepsis assessment
Proffitt and Hooper (2020)	To assess nurses’ perceptions towards the implementation of the 106 q-sofa assessment tool for sepsis	Quasi-experimental	III	14 ER nurses/ USA	The use of this tool led nurses to become more autonomous in making decisions related to sepsis, thus leading to prompt management of sepsis. Nurses perceived the lack of time to be a barrier to the implementation of the evidence-based treatment guidelines	**Weakness**: small sample size, single center **Strength**: employing a new sepsis screening tool
Rajan and Rodzevik (2021)	To explore the differences between ER nurses receiving an educational program on the early identification & management of sepsis & nurses not receiving the program	Quasi- experimental	III	22 ER nurses/ USA	Using sepsis standing orders combined with the educational program contributed to the early identification of sepsis and better quality of care provided.	**Weakness**: Small sample size & single center **Strength**:
Oliver (2018)	To assess the impact of EGDT on the early detection of sepsis in an ED	Quasi-experimental	III	63 patients with sepsis /USA	Revealed no significant differences in lactate measurement and blood culture collection but a decrease in time until antibiotic administration	**Weakness**: Single center, screening tool implemented over a short time period **Strength**: used valid and reliable tools
Burney et al. (2012)	To identify the barriers related to sepsis treatment	Descriptive-cross sectional	VI	101 ER nurses/ USA	Shortage of nurses, unavailability of ICU beds and limited physical space in were the most reported barriers to sepsis treatment	**Weakness:** single center and used self-report questionnaire **Strength:** provide detailed explanation about the barriers
Edwards & Jones (2021)	To examine nurses’ levels of knowledge, attitude, and skills related to sepsis management	Descriptive-cross sectional	VI	98 acute medical-surgical nurses/ UK	Nurses incorrectly answered the questions related to knowledge of sepsis and demonstrated positive attitudes.	**Weakness:** used self-report questionnaire **Strength**: used multi-settings
Steinmo el al. (2015)	To explore the effect of using behavioral science tools to modify the existing quality improvement guidelines for “Sepsis Six” implementation	Qualitative	VI	19 ER nurses, 12 ER doctors, 2 midwives and 1 healthcare assistant/ UK	Using behavioral science tools was feasible to modify the existing quality improvement guidelines for “Sepsis Six” implementation. The tools are compatible with the currently used pragmatic approach.	**Weakness**: fatigue was a threat to internal validity. **Strength**: used multi-settings and detailed face to face interviews
Giuliano et al. (2005)	to examine nurses’ understanding of clinical practice related to assessment of sepsis as well as their knowledge of diagnostic criteria for sepsis	Descriptive-cross sectional	VI	517 nurses& 100 physicians/ USA	The majority of participants routinely use the findings of PAP, Bp, O2 Sat, and ECG to assess and manage sepsis	**Weakness:** used self-report questionnaire **Strength**: large sample
Ferguson et al. (2019)	To assess the effectiveness of QI initiative in improving the early assessment and management of sepsis	Retrospective cohort	IV	106,220 patients with sepsis from a medical center in Seatle/USA	The implementation of QI improved ER sepsis bundle adherence by 33.2%, decreased sepsis-related RRT calls by 1.35% & in-hospital sepsis-related mortality rate by 4.1% (p<0.001)	**Weakness**: conducted in one site **Strength**: very large sample size
Giuliano et al. (2010)	To examine the difference in mean times required for sepsis recognition and treatment initiation between nurses exposed to 2 different monitor displays in response to simulated case scenarios of sepsis	Quasi-experimental	III	75 critical care nurses/ USA	mean times required for sepsis recognition and treatment initiation were shorter nurses exposed to EBM.	**Weakness**: screening tool implemented over a short time period & pilot study. **Strength**: used control group and random assignment
Kabil et al. (2021)	To explore ER nurses’ experiences of initiating early goal-directed fluid resuscitation in patients with sepsis	Qualitative	VI	10 ER nurses/ Australia	participating nurses identified different factors limiting the prompt initiation of early goal-directed fluid resuscitation, some challenges to the clinical practice of sepsis, and solutions to these challenges. Most nurses suggested incorporating nurse-initiated early goal-directed fluid resuscitation for patients with sepsis.	**Weakness**: limited generalizability of findings & interpretation bias **Strength**: used detailed face to face interviews

USA: United States of America; UK; United Kingdom; ACS: acute care settings; ER: emergency room; ICU: intensive care units; SIRS: Systematic Inflammatory Response Syndrome; KAP: knowledge, attitudes, and practice; qSOFA: Quick Sequential Organ Failure Assessment; EGDT; Early Goal-Directed Therapy; NDS: Nurse Driven Sepsis Screening tool; SIRS: Sepsis Inflammatory Response; MEW-S: Modified Early Warning Score; LRTI: Lower respiratory tract infection; IQ: Quality Improvement; EBM: Enhanced Bedside Monitor; RRT: rapid response team.

### Ethical considerations

There was no need to obtain ethical approval to conduct this systematic review since no human subjects were involved.

### Quality assessment and data synthesis

A quality assessment of the selected studies was performed independently by two researchers based on the guidelines of Melnyk and Fineout-Overholt [14]. Disagreements between the two researchers (MR, DB) were identified and resolved through a detailed discussion held during a face-to-face meeting. For complicated cases, the researchers (MR, DB) requested a second opinion from a third researcher (AH). According to the guidelines of Melnyk and Fineout-Overholt [14], twelve of the studies were at level 3 in terms of quality, four studies at level 5, and nine studies at level 6.

A qualitative synthesis was performed to synthesize the findings of the reviewed studies. The following steps were applied throughout the process of data synthesis:

1. The data in the selected studies were assessed, evaluated, contrasted, compared, and summarized in a table (Table 2). This data included the design, purpose, sample, main findings, strengths/limitations, and level of evidence for each of the studies.
2. The similarities and differences between the main findings of the selected studies were highlighted.
3. The strengths and limitations of the reviewed studies were discussed.

## Results

### Description of the selected studies

Most of the reviewed studies were conducted in Western countries [9, 11, 12], with only one study conducted in Eastern countries [1], and two in Middle-Eastern countries [15, 16]. The detailed geographical distribution of the studies and other characteristics are described in Table 2.

### Nurses’ knowledge, attitudes, and practices

Nine of the selected studies assessed nurses’ knowledge and attitudes related to sepsis assessment and management in critically ill adult patients [1, 9, 12, 15, 17–21] **(**Table 3**)**. Nucera et al. [18] found that ICU nurses had poor attitudes towards blood culture collection techniques and timing and poor levels of knowledge related to the early identification, diagnosis, and management of sepsis. For example, the majority of nurses reported that there is no need to sterilize the tops of culture bottles, and there is no specific time for specimen collection [18]. However, the participating nurses reported good levels of knowledge related to blood culture procedures and the risk factors for sepsis. Similarly, R. J. Roberts et al. [19] found the participating nurses to have good knowledge of septic shock and good attitudes toward the initiation of antibiotics for critically ill adult patients with sepsis. Only two studies assessed nurses’ practices related to sepsis assessment and management [15, 19]. For example, in the study of R. J. Roberts et al. [19], 40% of the nurse participants reported that they were aware of the importance of initiating antibiotics and IV fluid within one hour of septic shock recognition [20]. Also, Yousefi et al. [15] found the participating nurses to have good practices related to sepsis assessment and management.

**Table 3:** Knowledge, attitudes, and practices related to sepsis assessment and management.

Study	Knowledge (Mean Score, interpretation)	Attitudes (Mean Score, interpretation)	Practices (Mean Score, interpretation)
Van den Hengel et al. (2016)	15.9±3.21, above average	N/A	N/A
Rahman et al. (2018)	MNR, Moderate	*21–27, neutral	N/A
Storozuk et al. (2019)	^¥^51.8%, Poor	N/A	N/A
Harley et al. (2019)	MNR, Poor	N/A	N/A
Nucera et al. (2018)	MNR, Good	*51–75, poor	N/A
R.J. Roberts et al. (2017)	MNR, Good	N/A, positive	MNR, good
Yousefi et al. (2012)	64.5±5.21, MNR	73±4.51, MNR	81±4.31, MNR
Edwards & Jones (2021)	40.8%, Poor	25±2.97, positive	N/A
Giuliano et al. (2005)	MNR	N/A	N/A

*A range of the score reported

¥ a percentage of correct answers reported; MNR: Measured but not reported; N/A: Not Applicable

### Barriers to and facilitators of sepsis assessment and management

The reviewed studies identified three types of barriers to the early identification and management of sepsis, namely patient-, nurse-, and system-related barriers (Table 4). Meanwhile, only nurse- and system-related facilitators were reported in the reviewed studies. The most-reported barriers and facilitators were system-related. The reported barriers included (a) the lack of written sepsis treatment protocols or guidelines adopted as hospital policy [22, 23]; (b) the complexity and atypical presentation of the early symptoms of sepsis [19]; (c) nurses’ poor level of education and clinical experience [1, 12]; (d) the lack of sepsis educational programs or training workshops for nurses [22, 23]; (e) the high comorbid burden among patients with sepsis, which complicates the critical thinking process of sepsis management [19]; (f) nurses’ deficits in knowledge related to sepsis treatment protocols and guidelines [22–24]; (g) the lack of mentorship programs in which junior nurses’ actions/activities are strictly supervised by experienced nurses [17, 23]; (h) heavy workloads or high patient-nurse ratios [22]; (i) the shortage of well-trained and experienced physicians, particularly in EDs [19, 22, 23]; (j) the lack of awareness related to antibiotic use for patients with sepsis [19, 22]; (k) the lack of IV access and unavailability of ICU beds [25]; (l) the non-use of drug combinations for the treatment of sepsis [22, 26, 27], and (m) poor teamwork and communication skills among healthcare professionals [22, 26]. Only three facilitators of sepsis assessment and management were identified in the reviewed studies. These facilitators were (1) nurses’ improved confidence in caring for patients with sepsis, (2) increased consistency in sepsis treatment, and (3) positive enforcement of successful stories of sepsis management [22, 27].

**Table 4:** The barriers to and facilitators of sepsis assessment and management.

Barriers		
Patient-related barriers	Nurse-related barriers	System-related barriers
• Complexity and atypical presentation of the early symptoms of sepsis • High comorbid burden among patients with sepsis, which complicates the critical thinking of sepsis management	• Nurses’ poor level of education and clinical experience • Nurses’ knowledge deficits regarding the protocols and guidelines for the treatment of sepsis • Lack of awareness related to antibiotic use for patients with sepsis • Poor teamwork and communication skills among healthcare professionals	• Lack of written sepsis treatment protocols or guidelines adopted as hospital policies • Lack of sepsis educational programs or training workshops for nurses • Lack of mentorship programs in which junior nurses’ actions/activities are strictly supervised by experienced nurses • Heavy workloads or high patient-nurse ratios • Shortage of well-trained and experienced physicians, particularly in EDs • Lack of IV access and unavailability of ICU beds • Non-use of drug combinations for sepsis treatment
**Facilitators**		
Nurse-related		System-related
• Nurses’ improved confidence in caring for patients with sepsis		• Enhanced consistency in sepsis treatment • Positive enforcement of successful stories of sepsis management

### Measurement tools of sepsis-related knowledge, attitudes, and practices

One of the reviewed studies used a Knowledge, Attitudes, and Practice (KAP) questionnaire developed according to the Surviving Sepsis Campaign (SSC) guidelines [15] to measure nurses’ knowledge, attitudes, and practices related to sepsis assessment and management. Meanwhile, eight studies [1, 9, 12, 17–21] used self-developed questionnaires based on the literature and SSC guidelines and validated by expert panels. Details of these measurement tools and their psychometric properties are summarized in Table 5.

**Table 5:** A summary of the measurement tools and their psychometric properties.

Study	Name of the tool	Measured variable(s)	Description of the tool	# of items	Total score	Validity	Reliability*	Piloted
Van den Hengel et al. (2016)	Self-developed questionnaire	Knowledge of sepsis and SIRS criteria	General information about sepsis, SIRS, protocol, treatments, & case studies	35	29	Validated by expert panel	0.53	No
Oliver (2018)	Self-developed questionnaire	knowledge & practices related to antibiotic administration for sepsis	Information about sepsis management protocol & barriers to rapid antibiotic administration	NR	NR	NR	NR	Yes
Rahman et al. (2019)	Self-developed questionnaire	Knowledge & attitudes towards sepsis	Questions on the indicators of SIRS, sepsis criteria, case scenarios, and attitudes towards the early identification and management of sepsis	39	39	Face & content validity were assessed	0.86	Yes
Storozuk et al. (2019)	Self-developed questionnaire	Knowledge of sepsis	Questions about the signs & symptoms of sepsis, sepsis criteria, definition of sepsis, at- risk patients, & treatment	225	NR	NR	NR	Yes
Harley et al. (2019)	Self-developed questionnaire	Knowledge of sepsis	Questions on sepsis, sepsis criteria, SIRS, q SOFA, nursing role, & barriers to the early identification of sepsis	22	NR	Qualitative content analysis	N/A	No
Nucera et al. (2018)	Self-developed questionnaire	Knowledge & attitudes towards sepsis	Questions on the riskiest sepsis procedures, knowledge about the early identification of sepsis, & attitudes towards blood culture collection techniques	26	NR	NR	0.88	Yes
Edwards & Jones (2021)	Self-developed questionnaire	Knowledge, skills & attitudes towards sepsis	Closed & open-ended questions on nurses’ opinions and experiences regarding sepsis	24	NR	NR	NR	Yes
Yousefi et al. (2012)	KAP	Knowledge, attitudes, & practices related to sepsis	Questions about knowledge, attitudes, & practices related to sepsis	46	NR	Content validity was assessed	77–90.7	No
Giuliano et al. (2005)	Self-developed questionnaire	Knowledge of diagnostics criteria for sepsis	Questions about the physiologic parameters routinely used to assess for sepsis	20	NR	NR	Not measured	No

SIRS: Systematic Inflammatory Response Syndrome; KAP: knowledge, attitudes, and practice; NR: not reported; qSOFA: Quick Sequential Organ Failure Assessment

* Cronbach’s Alpha

### Interventions directed at improving nurses’ sepsis assessment and management

#### Educational programs

Only four of the selected studies examined the impact of educational programs on nurses’ knowledge, attitudes, and practices related to sepsis management and found significant improvements in nurses’ posttest scores (Table 6) [11, 15, 28, 29]. For example, Drahnak’s study [28] implemented an educational program developed by the authors and integrated with patients’ health electronic records (HER) and found significant improvements in nurses’ post-test nursing knowledge scores. Another educational program developed by the authors was implemented to improve ICU nurses’ knowledge, attitudes, and practices related to sepsis and found a significant improvement in posttest scores among the intervention group [15]. Another study was designed to examine the effectiveness of the Taming Sepsis Educational Program® (TSEP™) in improving nurses’ knowledge of sepsis [11]. A 15-minute structured educational session was developed to decrease the mean time needed to order a sepsis order set for critically ill patients through improving ER nurses’ knowledge about SSC guidelines and found that the mean time was reduced by 33 minutes among the intervention group [29].

**Table 6:** Sepsis education programs and simulations.

Study	Intervention/Control	Assessment Times	Measured Variable(s)	Differences in Posttest Scores Between Groups
Delaney et al. (2015)	*I: received 2 educational sessions. The first session consisted of 4 hours of online learning. The second session consisted of active participation in videotapes, high fidelity simulation, case scenarios, and debriefing sessions focusing on early sepsis assessment, care of septic patients, IHI bundles stages of sepsis, case studies, HLCC, & bundles of sepsis.	Post intervention	**Nurses’ knowledge on:** IHI bundles, SST, STEPS communication, & HLCC **Nurses’ competency:** Sepsis assessment Sepsis management EGDT initiation	+0.22 +0.32 +0.16 +0.02 +21.45 +24.16 +19.25
Yousefi et al. (2012)	I: received one PPT session (8 hour) about sepsis care, treatment, prevention, principles, nosocomial infections, and guidelines integrated with pamphlets. Assessed nurses’ knowledge, attitudes, and practices three times (pre-intervention, immediately post intervention, and three weeks post intervention). C: did not receive an educational program	Pre-intervention, immediately post intervention, & three weeks post intervention.	**Immediately Posttest** Knowledge: Attitudes: Practices: **3 weeks post intervention:** Knowledge Attitudes Practices	+21.0 +6.4 +7.6 +21.7 +10.1 +8.6
Drahnak (2016)	*I: received one session (30 minutes) with a voice-over slide presentation & role-play case study focusing on the pathophysiology of sepsis, risk factors for sepsis, SSC guidelines, case studies, and assessment of sepsis, integrated with HER	Before the educational program 1 month post intervention	Knowledge Attitudes Screening adherence: *Non-adherence *Partial adherence *Adherence	+56.22 -18.25 -31.74 +28.5 +3.4
Rajan et al. (2021)	I: received a structured educational session (15 minutes) focused on SIRS criteria, sepsis criteria, policy, sepsis screening tools, and sepsis standing order. C: did not receive an educational session	Post intervention	Time for sepsis identification	-33 minutes
Vanderzwan et al. (2020)	*I: received medium fidility simulation for 15 minutes. Nurses also received educational session about CLMS.	LMS & one week post simulation	Knowledge retention & competency related to the early identification & management of sepsis	outcomes improved after simulation
Giuliano et al. (2010)	I: exposed to EBM display which is a continuous visual display of combinations of recent data trends & parameters to promote early recognition of sepsis in response to a computer-simulated scenarioC: exposed to SBM display of 5 parameters including BP, ECG, PAP, CO, and O2 Sat which need to be intereprted by clinicans to meaningful data in response to a computer-simulated scenario • All partciapnts received educational program on sepsis assessment and management based on SSC guidelines	Immediately Pre-intervention & post intervention	Response time to the different monitor displays Time for sepsis recognition Times for SSC-recommended interventions initiation	Similar responses -1.32 minutes -1.33 minutes

*one group only; MNR: Measured but not reported, IHI bundles: Institute for Healthcare Improvement; HLCC; Health literacy and culture competency; EGDT; Early Goal Directed Therapy; SST: Staging sepsis Team; CLM: computerized Learning Management Systems; HER: Electronic Health Record; I: Intervention; C: Control; EBM: Enhanced Bedside Monitor; SBM: Standard Bedside Monitor; CDSS: Clinical Decision Support System

#### Simulation

Only two studies examined the effect of using simulation in improving the early recognition and prompt treatment of sepsis by critical care nurses (Table 6) [30, 31]. Vanderzwan et al. [30] assessed the effect of a medium-fidelity simulation incorporated into a multimodel nursing pedagogy on nurses’ knowledge of sepsis and showed significant improvements in six of the nine questionnaire items. While Giuliano et al. examined the difference in mean times required for sepsis recognition and treatment initiation between nurses exposed to two different monitor displays in response to simulated case scenarios of sepsis and showed a significant reduction in the mean times required for sepsis recognition and treatment initiation by those nurses who were exposed to enhanced bedside monitor (EBM) display [31].

#### Decision support tools

Four of the selected studies examined the effectiveness of decision support tools, adapted based on the SSC guidelines and the “sepsis alert protocol”, on the early identification and management of sepsis and confirmed the effectiveness of these tools (Table 7) [32–35]. The decision support tools used in three of the studies guided the nurses throughout their decision-making processes to reach effective assessment, high quality and timely management of sepsis, and, in turn, optimal patient outcomes [32, 33, 35]. However, no significant differences in the time of blood culture collection and antibiotic administration were reported between the intervention and control groups in the study of Delawder et al. [34].

**Table 7:** Sepsis decision-making support and screening tools and treatment protocols.

Study	Decision tool/sepsis protocol or tool	Description of the tool or protocol	Main effects on patient outcomes
Manaktala et al. (2017)	Sepsis Survilence Algorithim	The screening tool assesses sepsis clinical parameters (physical exam & lab test) & sends alam signals to nurses about positive findings.	Sepsis mortality rate was reduced by 53% & 30 day readmission was reduced from 19.08% to 13.21%. The tool sensitivity & specificity were 95% and 82%, respectively.
Amland et al. (2015)	Sepsis alert (Binary alarm system)	The tool consists of two steps. The first step is the detection of actual or potential sepsis, and the second is screening & stratification conducted within 15 minutes	89% of septic patients were detected by the alert system, & screening and stratification was completed for 75% of the cases within an hour from notification. The tool sensitivity was 94%.
Long et al. (2018)	User interface alert	User interface alert was designed for medical systems to a provide computer support system for decision-making related to sepsis	The tool enhanced reliability & specificty of patient data for detecting sepsis & provided an effective clinical decision support system for nurses to innititate sepsis assessment & management
Delawder et al. (2019)	Sepsis alert algorithim	Sepsis alert algorithim was designed to initiate full screening of sepsis when the nurse receives an electronic notification. This alert depends on the SIRS criteria & SSC guidelines	The alert algorithm can improve the time taken to implement sepsis guidelines except for antibiotics administration & blood culture collection. Mortality rate was decreased from 12.45% to 4.55%.
Proffitt et al. (2020)	qSOFA	It includes 2 parts, the first part being the assessment of potential infection & the second part being the assessment of Q-SOFA score, which is calculated based on GCS, systolic BP & RR.	The use of qSOFA led nurses to become more autonomous in making decisions related to sepsis management. The median time from ER admission to triage evaluation was reduced by 9 minutes.
McKinley et al. (2011)	TMH	If the patient had MAP<65 mmHg, LL >4 mmol/L, or U.O <0.5 mg/kg/hr, diagnostic tests, broad spectrum antibiotics, & fluid were initiated, and the lactate test was repeated after 4 hours. If the patient met two or more of the previous criteria, central venous line application would be added to the management plan	Time taken to initiate antibiotic administration, blood culture collection, & lactate level assessment & nurses’ compliance to sepsis treatment guidelines were improved, and the mortality rate declined with the use of TMH. The sensitivity & specificity of the TMH were 97%.
Oliver et al. (2018)	EGDT & NDS	The protocols are based on the SSC guidelines, and focus on blood culture, lactate measurement, and antibiotic administration	No significant differences in lactate measurement & blood culture collection were identified, but the time taken for antibiotic administration was improved.
Roney et al. (2020)	MEW-S	This tool was used for the early identification of at-risk patients based on the early signs of status deterioration according to body temperature, BP, RR, LOC, WBC, U.O & L.L.	MEW-S facilitated the early identification of sepsis & provision of timely management. The mortality rate declined by 24%.
Jacobs et al. (2020)	NDS	This tool was developed based on the SSC guidelines & had 4 steps: (1) measure lactate level, (2) take blood culture, (3) provide broad spectrum antibiotics, (4) administer 30 ml/kg crystalloid fluid if hypotensive & LL > 4 mmol/L, & (5) measure bilirubin, creatinine, GCS, MAP, RR, PT, PTT & platelets account.	The readmission rate was reduced from 36.28% to 25% 8 weeks after the NDS protocol, and compliance to the sepsis intervention protocol improved but with no effect on mortality rate.
Gyang et al. (2015)	NDS	Developed based on the SSC guidelines: (1) if the patient met >2 of the SIRS criteria>>> suspected sepsis; (2) if the patient screened >2 SIRS criteria >>> confirmed sepsis and presence of infection; (3) document findings in EHR & call physician	The tool sensitivity and specificity were 95.5% and 91.9%, respectively.
El-khuri et al. (2019)	EGDT	Developed based on the SSC guidelines depending on the following measurements: SIRS criteria, vital signs, U.O, O2 level, cardiac index, & continuous monitoring	There were no differences between the two groups in time and duration of vasopressor, antibiotic administration, or length of stay. However, the mortality rate was decreased from 47.6% to 31.7% with the implementation of EGDT.
Ferguson et al. (2019)	QI	Developed based on the SSC guidelines with few modifications: (1) administer 2 L of fluid instead of 30 ml/kg (2) apply it on patients with suspected infection, and (3) with 2 or more SIRS criteria	ER sepsis bundle adherence was improved by 33.2%, sepsis-related RRT calls was decreased by 1.35% & in-hospital sepsis-related mortality rate by was decreased 4.1% (p<0.001)

qSOFA: Quick Sequential Organ Failure Assessment; TMH: The Methodist Hospital; NDS: Nurse Driven Sepsis Screening tool; EGDT: Early Goal-Directed Therapy; SSC: Surviving Sepsis Campaign; SIRS: Sepsis Inflammatory Response; HER: Electronic Health Records; UO: Urine Output; O2: oxygen; Map: Mean Arterial Pressure; GCS: Glasgow Coma Scale; RR: Respiratory Rate; PT: Prothrombin Time; PTT: Partial Thromboplastin Time; LL: Lactate level; QI: Quality Improvement; RRT: rapid response team.

#### Sepsis protocols

Eight of the selected studies examined the effectiveness of sepsis protocols [24, 36–38] and sepsis screening tools [16, 39–41] for the early assessment and management of sepsis (Table 7). All of these articles revealed that the implementation of sepsis screening tools or protocols based on the SSC guidelines leads to the early identification and timely management of sepsis, as well as the improvement in nurses’ compliance to the SSC guidelines for the detection and management of sepsis. For example, in one study, patients who received Early Goal-Directed Therapy (EGDT) had a lower mortality rate as compared to patients who received usual care [16]. The sepsis screening tools and guidelines were also tested to examine their impact on some patient outcomes, and variabilities were identified. For example, the use of the Modified Early Warning Score (MEW-S) tool revealed no significant improvement in patient mortality rate [41]. In contrast, mortality rates were decreased by using the Nurse Driven Sepsis Protocol (NDS) [40], Quality Improvement (QI) initiative [38], and a computerized protocol [37]. In addition, nurses in the computerized protocol group had better compliance with the SSC guidelines than did nurses in the paper-based group [37]. One of the selected studies compared between a paper-based sepsis protocol and a computer-based protocol and found that antibiotic administration, blood cultures, and lactate level checks were conducted more often and sooner by nurses in the computerized protocol group [37]. Two of the selected studies used the EGDT as a screening tool for sepsis and found no significant differences in times of diagnosis, blood culture collection, or lactate measurements between the control and intervention groups [16, 24]. However, significant differences were found in the time of antibiotic administration in the study of Oliver et al. [24]. Although El-khuri et al. [16] revealed no significant differences in the time of antibiotic administration, the mortality rate among patients in the intervention group declined significantly.

## Discussion

Most of the reviewed studies focused on assessing critical care nurses’ knowledge, attitudes, and practices related to sepsis assessment and management, revealing poor levels of knowledge, moderate attitude levels, and good practices. Also, this review revealed that the three most common barriers to effective sepsis assessment and management were nursing staff shortages, delayed initiation of antibiotics, and poor teamwork skills. Meanwhile, the three most common facilitators of sepsis assessment and management were the presence of standard sepsis management protocols, professional training and staff development, and positive enforcement of successful stories of sepsis treatment. Moreover, this review reported on a wide variety of interventions directed at improving sepsis management among nurses, including educational sessions, simulations, screening or decision support tools, and intervention protocols. The impacts of these interventions on patient outcomes were also explored.

The findings of our review are consistent with the findings of previous studies which have explored critical care nurses’ knowledge related to sepsis assessment and management [42]. Also, recent studies conducted in different clinical settings support the findings of our review regarding nurses’ knowledge of sepsis. For example, a recent study conducted in a medical-surgical unit revealed that nurses had good knowledge of early sepsis identification in non-ICU adult patients [43]. The variations in nurses’ levels of knowledge related to sepsis assessment were attributed to variations in educational level and work environment (i.e., ICU vs. non-ICU).

The evidence indicates that the successful treatment of critically ill patients with suspected or actual sepsis requires early identification or assessment [44, 45]. Early assessment is a critical step for the initiation of antibiotics for patients with sepsis, leading to improved patient outcomes and a decline in mortality rates [44]. The current review also revealed the significant role of educational programs in improving nurses’ knowledge, attitudes, and practices related to the early recognition and management of sepsis. These findings are in line with the findings of another study, which tested the impact of e-learning educational modules on pediatric nurses’ retention of knowledge about sepsis [45]. The study revealed that the educational modules improved the nurses’ knowledge acquisition and retention and clinical performance related to sepsis management [45]. The findings of our review related to sepsis screening and decision support tools are in congruence with the findings of a previous clinical trial which assessed the impact of a prompt telephone call from a microbiologist upon a positive blood culture test on sepsis management [46]. The study revealed that this screening tool contributed to the prompt diagnosis of sepsis and antibiotic administration, improved patient outcomes, and reduced healthcare costs [46]. The findings of our review related to the effectiveness of educational programs in improving the assessment and management of sepsis were consistent with the findings of a recent quasi-experimental study. The study found that incorporating sepsis-related case scenarios in ongoing educational and professional training programs improved nurses’ self-efficacy and led to a prompt and accurate assessment of sepsis [47]. One of the interventions explored in this review was a simulation that facilitated decision-making related to sepsis management. The simulation was found to be effective in mimicking the real stories of patients with sepsis and proved to be a safe learning environment for inexperienced nurses before encountering real patients, increasing nurses’ competency, self-confidence, and critical thinking skills [48]. Also, a recent study showed that the combination of different interventions aimed at targeting sepsis assessment and management, including educational programs and simulation, may lead to optimal nurse and patient outcomes [49].

### Limitations

The present review has several limitations. There is limited variability in the findings of the reviewed studies in terms of the main variable, sepsis. Moreover, the review excluded studies written in languages other than English and conducted among populations other than critical care nurses. However, there may be studies written in other languages which may have significant findings not considered in this review. Further, only eight databases were used to search for articles related to the topic of interest, which may have limited the number of retrieved studies. Finally, due to the heterogeneity between the selected studies, a meta-analysis was not performed.

### Relevance to clinical practice

Our findings could help hospital managers in developing continuous education and staff development training programs on assessing and managing sepsis for critical care patients. Establishing continuous education, workshops, professional developmental lectures focusing on sepsis assessment and management for critical care nurses, as well as training courses on how to use evidence-based sepsis protocol and decision support and screening tools for sepsis, especially for critical care patients are highly recommended. Also, our findings could be used to development of an evidence-based standard sepsis management protocol tailored to the unmet healthcare need of patients with sepsis.

## Conclusion

To date, nurses remain to have poor to good knowledge of and attitudes towards sepsis and report many barriers related to the early recognition and management of sepsis in adult critically ill patients. The most-reported barriers were system-related, pertaining to the implementation of evidence-based sepsis treatment protocols or guidelines. Our review indicated that despite all educational interventions, no study has collectively targeted nurses’ knowledge, attitudes, and practices related to the assessment and treatment of sepsis using a multicomponent interactive teaching method. Such a method would aim to guide nurses’ decision-making and critical thinking step by step until a prompt and effective treatment of sepsis is delivered. Also, despite all available protocols and guidelines, no study has used a multicomponent intervention to improve health outcomes in adult critically ill patients. Future research should focus on sepsis-related nurse and patient outcomes using a multilevel approach, which may include the provision of ongoing education and professional training for nurses and the implementation of a multidisciplinary sepsis treatment protocol.

## Supporting information

S1 Checklist PRISMA 2020 checklist. (DOCX)

S1 File Search strategies. (PDF)
