# Supplementary material for: Sepsis assessment and management in critically Ill adults: A systematic review
Source: PLoS One. 2022 Jul 1;17(7):e0270711. doi: 10.1371/journal.pone.0270711 (PMC9249173; doi:10.1371/journal.pone.0270711)
Supplement: S1 File — (PDF) [file pone.0270711.s002.pdf]

Search terms used

1. ("critical care"[Mesh] OR "critically ill" [tiab] OR "intensive care"[tiab] OR "critical illness" [tiab]) AND ("knowledge"[Mesh] OR "awarness"[tiab] OR "perception"[tiab] OR "understanding"[tiab]) AND ("sepsis "[Mesh] OR "septic"[tiab] OR "septic shock"[tiab] OR "septicemia"[tiab]).

   4848 Articles

2. ("critical care"[Mesh] OR "critically ill" [tiab] OR "intensive care"[tiab] OR "critical illness" [tiab]) AND ("attitudes"[Mesh] OR "opinion"[tiab] OR "beliefs "[tiab] OR "thoughts"[tiab] OR " views" [tiab]) AND ("sepsis "[Mesh] OR "septic"[tiab] OR "septic shock"[tiab] OR "septicemia"[tiab]).

   985 Articles

3. ("critical care"[Mesh] OR "critically ill" [tiab] OR "intensive care"[tiab] OR "critical illness" [tiab]) AND ("practic"[tiab] OR "skills"[tiab] OR" stratigies"[tiab] OR "approaches"[tiab]) AND ("sepsis "[Mesh] OR "septic"[tiab] OR "septic shock"[tiab] OR "septicemia"[tiab]).

   5922 Articles

4. ("critical care"[Mesh] OR "critically ill" [tiab] OR "intensive care"[tiab] OR "critical illness" [tiab]) AND ("barriers "[tiab] OR "obstacles "[tiab] OR " challenges"[tiab] OR "difficulties "[tiab] " issues"[tiab] OR "problems "[tiab] OR "limitations"[tiab]) AND ("sepsis "[Mesh] OR "septic"[tiab] OR "septic shock"[tiab] OR "septicemia"[tiab]) .

   5799 Articles

5. ("critical care"[Mesh] OR "critically ill" [tiab] OR "intensive care"[tiab] OR "critical illness" [tiab]) AND("facilitators "[tiab] OR " motivators"[tiab] OR " enablers"[tiab]) AND ("sepsis "[Mesh] OR "septic"[tiab] OR "septic shock"[tiab] OR "septicemia"[tiab])

   28 Articles
